# Effects of Maternal Diet and Exercise during Pregnancy on Glucose Metabolism in Skeletal Muscle and Fat of Weanling Rats

**DOI:** 10.1371/journal.pone.0120980

**Published:** 2015-04-08

**Authors:** Mukesh Raipuria, Hasnah Bahari, Margaret J. Morris

**Affiliations:** 1 Department of Pharmacology, School of Medical Sciences, UNSW Australia, Sydney, NSW 2052, Australia; 2 Department of Human Anatomy, Faculty of Medicine and Health Sciences, Universiti Putra Malaysia, Selangor, Malaysia; University of Southampton, UNITED KINGDOM

## Abstract

Obesity during pregnancy contributes to the development of metabolic disorders in offspring. Maternal exercise may limit gestational weight gain and ameliorate these programming effects. We previously showed benefits of post-weaning voluntary exercise in offspring from obese dams. Here we examined whether voluntary exercise during pregnancy influences lipid and glucose homeostasis in muscle and fat in offspring of both lean and obese dams. Female Sprague-Dawley rats were fed chow (C) or high fat (F) diet for 6 weeks before mating. Half underwent voluntary exercise (CE/FE) with a running wheel introduced 10 days prior to mating and available until the dams delivered; others remained sedentary (CS/FS). Male and female pups were killed at postnatal day (PND)19 and retroperitoneal fat and gastrocnemius muscle were collected for gene expression. Lean and obese dams achieved similar modest levels of exercise. At PND1, both male and female pups from exercised lean dams were significantly lighter (CE versus CS), with no effect in those from obese dams. At PND19, maternal obesity significantly increased offspring body weight and adiposity, with no effect of maternal exercise. Exercise significantly reduced insulin concentrations in males (CE/FE versus CS/FS), with reduced glucose in male FE pups. In males, maternal obesity significantly decreased muscle myogenic differentiation 1 (MYOD1) and glucose transporter type 4 (GLUT4) mRNA expressions (FS vs CS); these were normalized by exercise. Maternal exercise upregulated adipose GLUT4, interleukin-6 (IL-6), tumor necrosis factor-alpha (TNF-α), and peroxisome proliferator activated receptor gamma coactivator 1 alpha (PGC1α) mRNA expression in offspring of dams consuming chow. Modest voluntary exercise during pregnancy was associated with lower birth weight in pups from lean dams. Maternal exercise appeared to decrease the metabolic risk induced by maternal obesity, improving insulin/glucose metabolism, with greater effects in male than female offspring.

## Introduction

Obesity is increasing worldwide across all age groups and is associated with a range of adverse outcomes. In the United States, more than 60% of women are overweight or obese at the time of conception [1]. Overweight and obese women are at increased risk of several pregnancy complications, including gestational diabetes mellitus, hypertension, preeclampsia, cesarean delivery, and postpartum weight retention [2]. Similarly, fetuses of obese mothers are at increased risk of prematurity, stillbirth, congenital anomalies, macrosomia with possible birth injury, and childhood obesity [3–5]. Maternal obesity has been associated with increased local and systemic inflammation; adipose tissue, placenta and vascular endothelial tissue are potential sources of inflammatory mediators during pregnancy [6].

Obese parents are more likely to have obese children, with many studies exploring the link between maternal body mass index (BMI) and offspring BMI. Maternal obesity and weight gain during pregnancy are related to higher BMI in childhood and subsequent obesity in adulthood [7]. Maternal obesity and excessive gestational weight gain are linked with greater weight gain in early infancy and higher risk of early childhood obesity [8]. Further, body weight during early childhood (kindergarten age) is a strong predictor of the development of later childhood obesity. Maternal obesity is also associated with insulin resistance and hyperinsulinemia in offspring [9,10].

Maternal obesity has been widely studied in rodent models where a maternal cafeteria or high fat (HF) diet during pregnancy has been shown to induce obesity, and insulin and leptin resistance in offspring [11–14]. Skeletal muscle plays a key role in systemic insulin sensitivity and in glucose uptake. Maternal obesity is associated with dysregulation of skeletal muscle development and its metabolic function in offspring, most probably due to altered insulin signaling [12,15]. Our lab demonstrated negative effects of maternal obesity [13] and postnatal overnutrition on glucose and lipid metabolism in the muscle of offspring [16]. Adipose tissue plays a decisive role in the genesis of obesity by regulating lipid homoeostasis and secreting hormones. In male rat offspring at postnatal day (PND)19, maternal obesity enhanced adipose tissue differentiation and upregulated lipogenic genes [17]. Adult offspring of obese dams showed significant increases in adipose tissue triglycerides, and an increase in enzymes involved in lipogenesis in adipose tissue [18].

Previously we found that voluntary exercise by offspring, either immediately post weaning or later in life, had beneficial effects, ameliorating maternal obesity induced metabolic disorders [19–21]. Exercise during gestation may be useful to limit gestational weight gain. Exercise is known to improve glucose and insulin homeostasis. In clinical studies exercise has been shown to have anti-inflammatory effects and improve insulin sensitivity by the upregulation of glucose transporter type 4 (GLUT4) expression in skeletal muscle [22]. In patients with type 2 diabetes, exercise training increased GLUT4 expression in adipose tissue [23]. Animal studies have recently begun to examine the effects of maternal exercise but the underlying molecular mechanisms are not clear [24,25].

The goal of the present study was to examine whether the detrimental effects of maternal obesity in offspring could be reduced by voluntary exercise by the mother, prior to and during pregnancy. We hypothesized that voluntary exercise during pregnancy would exert beneficial effects on glucose and lipid metabolism and would limit the detrimental impact of maternal obesity in offspring. To gain mechanistic insight, we wished to explore the effects of maternal exercise prior to and during pregnancy, on lipid and glucose metabolism in offspring of both lean and obese dams by measuring expression of key genes related to glucose, lipid metabolism and inflammation in fat and muscle at PND19. Sirtuin 3 (SIRT3) is a mitochondrial sirtuin and its expression increases with exercise and caloric restriction, while it decreases upon long-term high fat feeding [26,27]. Recently, it has been shown that obesity during gestation was associated with reduced SIRT3 expression and mitochondrial function in mothers [28] and in rat offspring at weaning [29]. However, an effect of maternal exercise on SIRT3 expression in the offspring, and any interaction with maternal obesity is not known. Thus, here we measured markers related to glucose metabolism and inflammation in muscle and fat. In muscle expression of GLUT4, peroxisome proliferator activated receptor gamma coactivator 1 alpha (PGC1-α), SIRT3, uncoupling protein 3 (UCP3), myogenic differentiation 1 (MYOD1), tumor necrosis factor-alpha (TNF-α), and interleukin-6 (IL-6) were measured. Additionally, we measured several of these markers in white adipose tissue (WAT).

## Materials and Methods

### Animals, Diet and Exercise

All procedures involving animals were approved by the Animal Care and Ethics Committee, UNSW, Australia (Approval No. 11/104B). Young female Sprague Dawley rats (n = 56) aged 6 weeks and weighing 160–170 grams were obtained from the Animal Resources Centre, Perth, Australia. Animals were maintained at 20 ± 2ºC on a 12–12 hour light-dark cycle. Rats were divided into two groups and fed either standard chow (Gordon’s Stockfeeds, NSW, Australia) or commercial HFD *ad libitum* supplemented with a selection of western foods (pies, cakes, dim sims, biscuits), yielding chow (C; n = 24) and HFD (F; n = 32) groups. The HFD was group offered three types of chow. In addition to regular chow, two types of commercially available high fat pellet diets; SF03–020, 43% of total energy from fat (20 MJ/kg, 23% fat, 19.4% protein, 44.9% carbohydrate), SF03–002, 59% of total energy from fat (22.8 MJ/kg, 36% fat, 19.4% protein, 40% carbohydrate), Specialty feeds, Australia. The nutritional information of the western food items is provided in S1 Table. After 6 weeks, rats were housed 2 per cage and half of each dietary group were given continuous access to a running wheel in their home cage (CE or FE) while the remainder were provided with a locked wheel, and thus remained sedentary (CS or FS). Mating (with males fed standard chow) began after 10 days of exercise and the pre-pregnancy diet was maintained throughout gestation/lactation. After confirmation of pregnancy they were singly housed until the end of the experiment. After delivery, all dams and pups were transferred to a normal cage with no running wheel. The average litter size and male to female ratios were not significantly different across the groups. At PND 1, litters were adjusted to 12 pups per mother. Two male and two female pups from each mother were culled at weaning (PND 19), under nonfasted conditions.

### Plasma and tissue collection

Offspring (PND19) and dams (4 weeks post-partum) were anaesthetized (ketamine/xylazine 180/32 mg/kg). Blood was collected via cardiac puncture for glucose (Accu-Check glucose monitor, Roche Diagnostics Australia), plasma triglycerides and hormone analysis. After decapitation, skeletal muscle (gastrocnemius and anterior tibialis) and WAT [retroperitoneal (Rp), gonadal and visceral] were dissected and weighed. Gastrocnemius muscle and RpWAT were snap frozen and stored at -80°C prior to mRNA measurement.

Plasma triglycerides were analyzed colorimetrically using commercially available triglyceride reagent (Roche, Australia) and standard (Sigma, USA). Plasma leptin and insulin concentrations were quantified using commercial radioimmunoassay kits (Millipore Corporation, USA).

### Quantitative real-time polymerase chain reaction (qRT-PCR)

RNA was extracted using Tri-reagent (Sigma, USA) and stored at -80°C. One µg RNA was reverse transcribed to complementary deoxyribonucleic acid (cDNA) (Omniscript Reverse Transcription kit; Qiagen, Chatsworth, California, USA). Preoptimized Taqman probe/primers (Applied Biosystems, Foster City, California, USA) including GLUT4, TNF-α, IL-6, and PGC1α were determined in RpWAT, while MYOD1, GLUT4, IL-6, TNF-α, UCP3, PGC1α and SIRT3 mRNA were determined in gastrocnemius muscles (S2 Table). Two housekeeper genes were selected from a set of 6 genes; beta actin (ActB), glyceraldehyde 3-phosphate dehydrogenase (GAPDH), peptidylprolyl isomerase A / cyclophilin A (PPIA), hypoxanthine phosphoribosyltransferase 1 (HPRT1), tyrosine 3-monooxygenase / tryptophan 5-monooxygenase activation protein zeta (YWHAZ) and beta 2 microglobulin (B2M) that displayed the most stability and least variation across treatment groups in individual samples using Normfinder (Version 0.953). mRNA expression of target genes in the gastrocnemius muscle were compared to YWHAZ and B2M. In RpWAT, target genes were compared to HPRT1 and B2M. The geometric mean was used to obtain Ct values of the housekeeper genes [30] and gene expression values were calculated using the ^ΔΔ^CT method [31]. A separate pooled sample from the untreated control group was arbitrarily assigned as calibrator.

### Statistical Analysis

Statistical analyses were performed using SPSS 20.0 Windows (SPSS Inc., Chicago, Il). Results are expressed as mean ± SEM. Weekly body weight and glucose tolerance test of dams were analyzed by two-way mixed ANOVA with repeated measures followed by post hoc least significance differences (LSD) test. Final body weight, blood glucose, plasma insulin, triglycerides and organ weights were analyzed by two-way ANOVA (maternal diet x maternal activity), followed by post hoc LSD. Running distance was analyzed by Student’s unpaired t-test. The mRNA expression data in muscle and WAT from male and female offspring were analyzed separately by two-way ANOVA (maternal diet x maternal activity), followed by post hoc LSD.

## Results

### Maternal phenotype, exercise level and hormonal status

Total 24 hour energy intake, measured just prior to mating was 244.9 ± 10.9 and 486.1 ± 18.7 kJ/ rat in the lean and obese dams respectively. The obese dams consumed 60% more energy than the lean group. Lean (CE) and obese (FE) dams exercised to a similar degree, showing modest exercise levels as judged by the total running distance until the end of pregnancy, Table 1. While FE appeared to run slightly less than CE, this failed to reach significance (*P* >.05). Distance run generally declined as pregnancy progressed. When distance run during the first 4 weeks was compared, it was also similar in the two groups (FE vs CE).

**Table 1:** Maternal body weight, fat and muscle mass, blood glucose, plasma insulin, triglyceride concentrations and maternal exercise level.

	Lean dams		Obese dams	
	CS (10)	CE (7)	FS (11)	FE (7)
Body Weight (g)	306.2 ± 8.4	301.4 ± 9.4	358.6 ± 10.5**	358.2 ± 5.9**
Retroperitoneal WAT (g)	1.1 ± 0.1	1.0 ± 0.1	3.8 ± 0.34**	3.5 ± 0.4**
Total WAT (g)	4.4 ± 0.3	4.0 ± 0.8	10.1 ± 0.6**	9.4 ± 0.9**
Total muscle (g)	0.71 ± 0.05	0.82 ± 0.05	0.82 ± 0.02	0.93 ± 0.02
Blood glucose (mmol.l^-1^)	6.3 ± 0.3	6.5 ± 0.3	6.5 ± 0.2	6.6 ± 0.3
Plasma insulin (ng.ml^-1^)	0.45 ± 0.2	0.49 ± 0.2	0.79 ± 0.2**	0.91 ± 0.3**
Plasma TG (mmol.l^-1^)	8.2 ± 1.3	5.7 ± 0.7	22.6 ± 4.3**	14.2 ± 2.6
Exercise level (km)	-	8.1 ± 4.5	-	5.1 ± 1.5

Body weight, retroperitoneal WAT, total WAT, muscle mass, blood glucose, plasma insulin and triglyceride concentrations of lean and obese dams at 4 weeks post-partum. Total fat represents the sum of retroperitoneal, gonadal and visceral fat pads. Muscle mass represents the sum of gastrocnemius and tibialis anterior muscles. Data are expressed as mean ± SEM. The first letter describes maternal diet; chow (C) or HFD (F) and the second letter describes maternal activity; sedentary (S) or exercise (E). Data were analyzed by two-way ANOVA followed by post hoc LSD comparison.

**P < .01, maternal HFD effect.

At the time of mating HFD fed dams were 19% heavier compared to lean dams (FS vs CS). At 4 weeks postpartum, HFD feeding was associated with significantly increased body weight regardless of dams’ activity (*P* < .01, Table 1) with no significant effect of exercise. Maternal HFD consumption was associated with higher adiposity, with more than a trebling and doubling of RpWAT and total WAT masses respectively, in sedentary and exercised dams as compared to chow fed dams (*P*< .01, Table 1). No significant effect of diet or exercise on the sampled muscle mass was observed.

**Fig 1:**
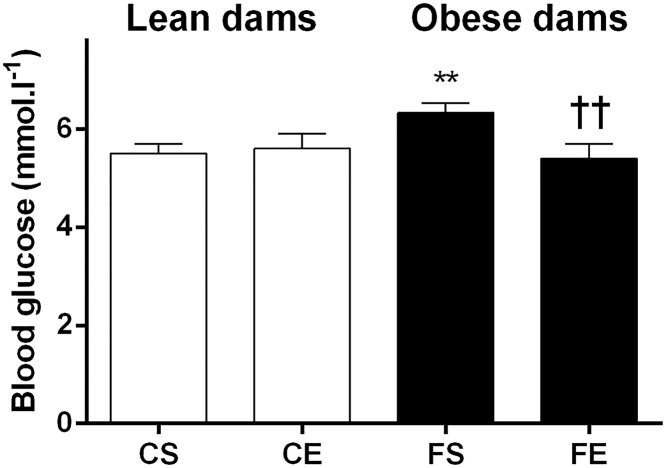
Maternal blood glucose concentration during the lactation period, at postpartum day 12. Data are expressed as mean ± SEM. The first letter describes maternal diet; chow (C) or HFD (F) and the second letter describes maternal activity; sedentary (S) or exercise (E). Data were analyzed by two-way ANOVA followed by post hoc LSD. **P< .01 maternal HFD effect; ^††^ *P* < .01 maternal exercise effect.

Maternal exercise during pregnancy had beneficial effects in obese dams by maintaining blood glucose at similar levels to control dams during the lactation period (postpartum day 12, Fig. 1). There was no significant difference in blood glucose level at 4 weeks postpartum across groups (Table 1). Plasma insulin concentrations were significantly increased by HFD consumption in obese dams regardless of their exercise activity (*P* < .01, Table 1). No effect of exercise on plasma insulin was observed. Maternal HFD consumption was associated with hyperlipidemia; plasma triglyceride levels were significantly increased in sedentary obese dams with a reduction in obese exercising dams that did not reach significance (FE vs FS, *P* < .06, Table 1).

#### Effects of maternal obesity and exercise during pregnancy on offspring at PND1 and 19 Body weight

**Fig 2:**
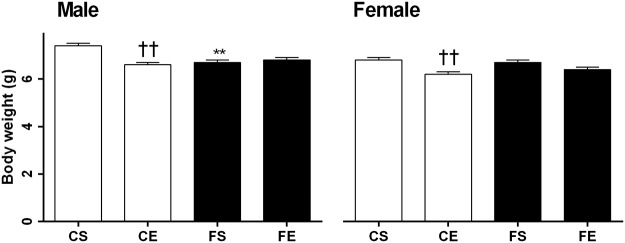
Body weight of male and female pups at postnatal day 1. Data are expressed as mean ± SEM. The first letter describes maternal diet; chow (C) or HFD (F) and the second letter describes maternal activity; sedentary (S) or exercise (E). Data in male and female offspring were analyzed separately by two-way ANOVA with maternal diet and maternal activity as factors, followed by post hoc LSD. **P < .01 maternal HFD effect; ^††^P < .01 maternal exercise effect.

Maternal obesity had sex specific effects on body weight at PND1; male pups from obese dams were significantly lighter (FS vs CS, *P* < .01, Fig. 2), with no significant difference in female pups. At PND1, lean exercised dams had lighter male and female pups with no effect of exercise in those from obese dams (CE versus CS, *P* < .01, Fig. 2). At PND19 while body weight of CE pups remained slightly reduced (~8%) compared to CS, this was not significant. Maternal obesity significantly increased both male and female pups’ body weight, regardless of maternal activity (FS vs CS and FE vs CE, *P* < .01, Table 2). Male and female body weights were similar at this age.

**Table 2:** Offspring body weight, visceral fat, total fat, muscle mass, blood glucose, plasma insulin and triglyceride concentrations at PND19.

		Male				Female		
	CS (17)	CE (14)	FS (16)	FE (12)	CS (18)	CE (13)	FS (21)	FE (12)
Body weight (g)	33.4 ± 1.0	30.0 ± 0.6	49.7 ± 2.7**	49.8 ± 2.6 **	31.5 ± 0.9	28.9 ± 0.5	47.3 ± 1.4*	46.8 ± 1.4*
Visceral fat (mg)	125.4 ± 5.3	107.5 ± 3.9^†^	229.0 ± 17.3**	250.0 ± 13.1 **	106.7 ± 6.3	90.3 ± 7.1	234.6 ± 10.8*	213.5 ± 13.7*
Total WAT (mg)	283.6 ± 24.8	223.1 ± 19.5	675.6 ± 66.9**	713.9 ± 41.6**	143.5 ± 8.8	122.3 ± 7.8	385.8 ± 2.4**	387.0 ± 22.9**
Total muscle (mg)	161.9 ± 7.6	137.3 ± 4.4	221.8 ± 19.6**	236.3 ± 23.9**	149.0 ± 7.0	141.8 ± 10.4	197.7 ± 14.4**	209.7 ± 15.9**
Blood glucose (mmol.l^-1^)	7.6 ± 0.3	7.8 ± 0.2	8.7 ± 0.3**	7.9 ± 0.4^†^	7.6 ± 0.1	7.6 ± 0.1	8.5 ± 0.2**	8.2 ± 0.2**
Plasma insulin (ng.ml^-1^)	1.8 ± 0.2	1.3 ± 0.1^†^	1.7 ± 0.2	1.0 ± 0.1^**†**^	0.6 ± 0.1	0.7 ± 0.1	2.1 ± 0.2**	2.1 ± 0.2**
Plasma TG (mmol.l^-1^)	26.6 ± 2.7	20.8 ± 2.6^†^	27.1 ± 3.4	32.7 ± 4.7*	16.1 ± 1.2	15.0 ± 2.9	22.6 ± 2.3	21.9 ± 1.9

Body weight, visceral fat, total WAT, muscle mass, blood glucose, plasma insulin and triglyceride concentrations in male and female offspring at PND19.

Total fat represents the sum of retroperitoneal, gonadal and visceral fat pads. Muscle mass represents the sum of gastrocnemius and tibialis anterior muscles. Data are expressed as mean ± SEM. The first letter describes maternal diet; chow (C) or HFD (F) and the second letter describes maternal activity. Data in male and female offspring were analyzed separately by two-way ANOVA with maternal diet and maternal activity as factors, followed by post hoc LSD.

*P < .05;

**P < .01 maternal HFD effect;

†P < .05 maternal exercise effect

#### Fat, muscle mass, blood glucose, plasma insulin and triglyceride concentrations

At PND19, pups from the obese dams had greater skeletal muscle mass and more than double WAT mass as compared to pups from lean dams (Table 2). Maternal exercise significantly reduced visceral fat mass in male pups from lean exercised dams (CE vs CS, *P* < .05, Table 2). No significant effect of maternal exercise on total muscle or WAT mass was observed when expressed as net mass (Table 2) or percent body weight (data not shown).

Maternal obesity and exercise had sex-specific effects on the blood glucose, plasma insulin and triglyceride concentrations of offspring. In males, maternal obesity was associated with higher blood glucose concentrations and maternal exercise had moderate beneficial effects, with reduced blood glucose in pups from obese dams. Conversely, maternal exercise had no effect on blood glucose concentrations in female pups (Table 2). Insulin concentrations were reduced in male pups from both lean and obese exercised dams (CE vs CS, FE vs FS, *P* < .05, Table 2) with no significant effect of maternal diet. On the other hand, in females, insulin concentrations were three times higher in pups of obese dams (FS vs CS and FE vs FS, *P* < .01, Table 2). There was no effect of maternal exercise on insulin level in female pups. In male pups, plasma triglycerides were significantly lower in pups from lean exercised dams, while triglycerides were moderately higher in pups from obese exercised dams (CE vs CS, FE vs CE, *P* < .05, Table 2). At PND19, neither maternal obesity nor exercise had any effects on plasma triglycerides in female pups.

#### Gene expression in skeletal muscle and adipose tissue from offspring at PND19

We next examined the expression of key determinants of glucose and lipid homeostasis in muscle and WAT. GLUT4 is the glucose transporter found primarily in the muscle and fat. In male offspring the expression of GLUT4 in the skeletal muscle was significantly downregulated by maternal obesity (FS vs CS, *P* < .01, Table 3) and it was normalized by maternal exercise (FE vs FS, *P* < .05, Table 3). A similar pattern was observed with the myogenesis related gene MYOD1; in males, its expression was significantly reduced by maternal obesity, and in obese dams’ offspring this was normalized by maternal exercise (FS vs CS, *P* < .01; FE vs FS, *P* < .05, Table 3). PGC1α, a transcriptional coactivator that regulates the genes involved in energy metabolism, was upregulated in male offspring by maternal exercise (CE vs CS, *P* < .05, Table 3). In males, mRNA expression of TNF-α, a marker of inflammation was significantly increased in the offspring from obese dams (FS vs CS, *P* < .05, Table 3). Maternal obesity and exercise during pregnancy appeared to have sex specific effects, and in this study, females were less affected. Unlike the males, there were no significant differences in GLUT4 or MYOD1 mRNA expression in female offspring. There was also no change in the expression of inflammatory cytokines; PGC1α, UCP3 or SIRT3, in skeletal muscle in response to either maternal obesity or exercise at PND19 (Table 3).

**Table 3:** Relative gene expression in gastrocnemius muscle of offspring at PND19.

	Male				Female			
	CS (17)	CE (14)	FS (16)	FE (12)	CS (18)	CE (13)	FS (21)	FE (12)
GLUT4	1.03 ± 0.06	0.96 ± 0.08	0.67 ± 0.03**	0.85 ± 0.05 ^**†**^	1.04 ± 0.09	1.16 ± 0.13	0.88 ± 0.06	0.97 ± 0.07
MYOD1	1.05 ± 0.12	0.77 ± 0.17	0.65 ± 0.09**	0.84 ± 0.16 ^**†**^	1.05 ± 0.10	1.21 ± 0.10	0.82 ± 0.09	1.01 ± 0.06
SIRT3	1.05 ± 0.10	0.98 ± 0.13	0.99 ± 0.13	0.84 ± 0.10	1.06 ± 0.11	0.96 ± 0.10	0.99 ± 0.07	1.06 ± 0.11
IL6	1.23 ± 0.20	0.96 ± 0.21	0.83 ± 0.11	0.85 ± 0.18	1.06 ± 0.12	1.40 ± 0.13	1.04 ± 0.11	0.86 ± 0.05
PGC1α	1.12 ± 0.12	1.52 ± 0.10^**†**^	1.09± 0.07	1.23 ± 0.11	1.09 ± 0.10	1.18 ± 0.07	1.02 ± 0.08	1.11 ± 0.06
UCP3	1.01 ± 0.12	1.22 ± 0.20	1.11 ± 0.10	1.31 ± 0.15	0.96 ± 0.06	1.20 ± 0.09	1.15 ± 0.09	1.23 ± 0.10
TNFα	1.20 ± 0.11	1.26 ± 0.15	1.67 ± 0.15*	1.38 ± 0.11	1.14 ± 0.16	1.47 ± 0.15	1.23 ± 0.10	1.30 ± 0.11
GLUT4	1.03 ± 0.06	0.96 ± 0.08	0.67 ± 0.03**	0.85 ± 0.05 ^**†**^	1.05 ± 0.10	1.21 ± 0.10	0.82 ± 0.09	1.01 ± 0.06

Relative gene expression in gastrocnemius muscle in male and female offspring at PND19. Data are expressed as mean ± SEM. The first letter describes maternal diet; chow (C) or HFD (F) and the second letter describes maternal activity. Data in male and female offspring were analyzed separately by two-way ANOVA with maternal diet and maternal activity as factors, followed by post hoc LSD.

*P < .05;

**P < .01 maternal HFD effect;

^†^P < .05 maternal exercise effect.

**Fig 3:**
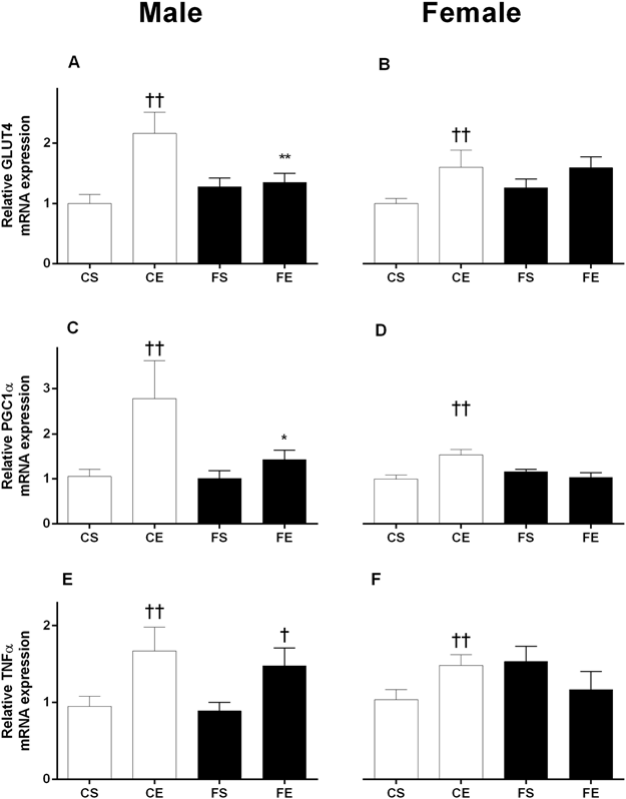
Relative gene expression in RpWAT of male and female offspring at PND19. Male: GLUT4 (A), PGC1α (C) and TNF-α (E), Female: GLUT4 (B), PGC1α (D) and TNF-α (F), gene expression. Data are expressed as mean ± SEM. The first letter describes maternal diet; chow (C) or HFD (F) and the second letter describes maternal activity; sedentary (S) or exercise (E). Data were analyzed by two-way ANOVA with maternal diet and maternal activity as factors, followed by post hoc LSD comparison. *P < .05; **P < .01 maternal diet effect; ^†^P < .05; ^††^P < .01 maternal exercise effect.

In RpWAT, the mRNA expression of GLUT4 and PGC1α were upregulated in male and female offspring born to lean exercised dams compared to sedentary dams, whereas no effect of exercise was observed in obese dams’ offspring (CE vs CS, *P* < .01, Fig. 3). For markers related to inflammatory signaling pathways, TNFα was significantly increased by exercise in male pups from both lean and obese dams (CE vs CS, FE vs FS, *P* < .05, Fig. 3). For IL-6 a similar pattern was observed however obesity appeared to dampen the impact of exercise on IL-6 mRNA in offspring of obese dams (CE 2.5±0.5 vs CS 0.87±0.2; FE 1.58±0.3 vs FS 0.87±0.1, P<0.05). In females, the mRNA expression of TNF-α was significantly increased in the pups from lean exercised dams (CE vs CS, *P* < .05, Fig. 3). As very low/inconsistent IL-6 expression was observed in RpWAT from females, these data are not shown.

## Discussion

In line with our previous work in rats [13,19,32], the findings of this study include significant effects of maternal obesity to increase body weight, fat and muscle mass, blood glucose, insulin and triglycerides in the offspring at PND19. In this study exercise by lean dams during pregnancy was associated with lower pup birth weight. Voluntary exercise by obese dams during pregnancy also improved glucose homeostasis in their offspring, with decreased plasma glucose and insulin concentrations in male pups at PND19. Plasma triglyceride, insulin concentrations and visceral fat mass were decreased in pups from lean exercised dams, with no significant effect on body weight. Hence maternal exercise had effects in offspring of both lean and obese dams. Another important observation was that these effects were sex specific and males appeared to benefit more from maternal exercise than females. In male offspring, exercise during pregnancy was able to decrease the metabolic risk conferred by maternal obesity by improving insulin and glucose metabolism.

### Diet and exercise effects in dams

As expected and in line with clinical studies [5,6,33], dams consuming HFD had significantly higher plasma insulin and more than doubled plasma triglycerides compared to lean dams at 4 weeks postpartum. We found beneficial effects of maternal exercise in these obese dams on glucose and triglyceride concentrations. Thus voluntary exercise during pregnancy was associated with lower blood glucose at PND12 and modestly reduced maternal triglyceride concentrations at 4 week postpartum (P = 0.06); interestingly the magnitude of the benefit appears similar in both lean and obese dams. This is in partial agreement with a study in albino Wistar rats which reported that maternal exercise reduced triglyceride, glucose and insulin concentrations at the end of lactation [25]. In our hands, maternal exercise had no lasting (4 week postpartum) effect on insulin and blood glucose levels, independent of maternal diet. This could be related to the modest exercise level of the dams; it is known that vigorous exercise favors negative energy and lipid balance to a greater extent than exercise of low to moderate level [34] and limited impact on metabolic measures was observed following low volume exercise in rats [35].

### Effects in offspring

Maternal diet and exercise during pregnancy had sex specific effects. In rodents variable effects of maternal obesity on birth weight have been reported, with most studies reporting no effect on birth weight [25,36,37]. In the present study, male pups from obese dams were born smaller, but as the dams consumed HFD during lactation, they gained weight and became heavier over time compared to offspring from lean dams such that at PND19, FS pups weighed 49% more than CS. Body weight early in life is a key indicator of risk of metabolic disorders in later life [38]. Babies who are born small and then show rapid growth may have increased risk of becoming obese in later life [39,40]. In accordance with our [13,16,19–21,41] and others [12,36,42] previous work, in the current study weanling pups from obese dams had a greater risk for metabolic disorders, with higher body weights, insulin, glucose, and triglyceride concentrations, and more than doubled WAT mass compared to pups from lean dams.

In humans, variable data on the effects of exercise during pregnancy on birth weight of newborns have been reported, with evidence for a decrease [43,44], increase [45] or no change [46]. The inconsistencies across studies could be related to several factors, including the type and level of exercise achieved during pregnancy. Interestingly, in the current study, exercise led to smaller birth weights in male and female pups of dams consuming the control diet. This is in line with studies where lean exercised dams had smaller babies [43,44]. These pups showed less growth than those from obese dams, and remained slightly lighter than pups from chow sedentary dams at PND19. It is not clear whether this low birth weight represents a positive adaptation. Rapid growth in infants is associated with faster linear growth and maturation, and is a risk factor for childhood or adult obesity [47]. Thus, further studies are recommended for understanding the effects of maternal exercise on growth in later life.

Clinical and animal data suggest that maternal obesity during pregnancy adversely affects offspring health in the short- and long-term, including increased risk of obesity development and co-morbidities such as type 2 diabetes, leading to premature death in adult offspring [48]. Hyperglycemia is a hallmark of diabetes, prediabetes and insulin resistance. In the current study we observed beneficial effects of maternal exercise on blood glucose levels in male offspring, normalizing the raised glucose induced by maternal obesity. In this cohort maternal obesity and exercise had sex specific effects on plasma insulin concentrations. Notably, only female offspring showed hyperinsulinemia in response to maternal obesity, with no effect of exercise. On the other hand, in males, maternal exercise reduced plasma insulin concentrations, with no effect of maternal diet. Thus, maternal exercise might be linked to reduced circulating blood glucose by increasing the rate of glucose uptake into skeletal muscles and other peripheral tissues. To investigate the molecular mechanisms, we examined the expression of key genes involved in glucose and lipid metabolism in the muscle and fat as these are major tissues involved in glucose and fatty acid metabolism.

GLUT4 and PGC1α are key regulators of energy metabolism in the muscle and fat, and the effects of exercise on GLUT4 have been widely studied. Exercise increases skeletal muscle glucose uptake and is associated with increased GLUT4 [49]. We previously found that maternal obesity downregulated GLUT4, with reduced mRNA expression [16] and protein content [41] in skeletal muscle of offspring from obese dams. Interestingly, here we found maternal exercise reduced plasma glucose and insulin concentrations in male offspring from obese dams and this was associated with an increased mRNA level of GLUT4 in skeletal muscle, suggesting enhanced glucose disposal in muscle [50], although further work would be required to confirm this.

In the current study, we found diet and tissue specific effects of maternal exercise on GLUT4 expression in offspring. Exercise during pregnancy by lean dams led to improved GLUT4 expression in WAT. The expression of PGC1α, which stimulates GLUT4 expression and mitochondrial biogenesis [51,52], was also increased by maternal exercise in fat and muscle, suggesting exercise by lean dams had a programming effect to limit fat accumulation and increased oxidative capacity. Exercise and long-term training in rats led to increases in PGC1α mRNA expression in WAT [53–55]. Thus, it has been speculated that exercise by lean dams limited excess visceral fat accumulation and reduced circulating triglycerides [25] with improved glucose uptake and reduced insulin in offspring.

Myogenesis occurs only in late gestation and during the neonatal period in rodents and is regulated by myogenic factors that include myogenin and MYOD1 [56]. In line with our previous report in rats [16] and from other laboratories in sheep [57], here we report downregulation of the myogenic marker MYOD1 in muscle of male offspring born to obese dams. Reduced expression of MYOD1 may lead to a reduced number of myogenic cells and thus affect muscle development, thus, maternal malnutrition in pregnancy and lactation can affect the contractile properties of skeletal muscle in the offspring [15]. Exercise by obese dams during pregnancy reversed the maternal obesity induced downregulation of MYOD1 in male offspring. To our knowledge this is the first report of an effect of maternal exercise on MYOD1 expression in male offspring.

Neonates [58] and children [59] of overweight and obese women are at greater risk of systemic inflammation compared to those born to women with lower BMIs. Maternal obesity is linked to low-grade inflammation which leads to harmful, persistent effects in offspring, including predisposition to obesity and insulin resistance [57]. In this study, we found an increased gene expression of pro-inflammatory adipokines, TNF-α and IL-6 with maternal exercise in the WAT of male offspring with no effect of maternal diet. IL-6 is known to induce lipolysis and fat oxidation and is involved in glucose homeostasis during exercise. In addition, IL-6 has strong anti-inflammatory effects and may inhibit TNF-α induced insulin resistance [60].

In conclusion, maternal obesity in rats during pregnancy increased the health risks for both mother and offspring with increased body weight, adiposity, hyperglycemia and hyperinsulinemia. Modest voluntary exercise by obese dams prior to and during pregnancy had benefits in normalizing their blood glucose concentrations during the lactation window. Maternal exercise seems to have beneficial effects to limit the impact of maternal obesity in offspring by reducing plasma insulin and glucose concentrations at PND19. On the other hand, in lean dams exercise during pregnancy limited visceral fat deposits and improved glucose homeostasis at PND19. Further studies with different levels and duration of exercise during pregnancy are recommended for increasing our understanding of the mechanisms behind the beneficial effects of exercise at different time points.

## Supporting Information

S1 Table Dams western/cafeteria diet information. (DOCX)

S2 Table Taqman probe sequence used for real time PCR. (DOCX)
