# Supplementary material for: Effects of Maternal Diet and Exercise during Pregnancy on Glucose Metabolism in Skeletal Muscle and Fat of Weanling Rats
Source: PLoS One. 2015 Apr 8;10(4):e0120980. doi: 10.1371/journal.pone.0120980 (PMC4390148; doi:10.1371/journal.pone.0120980)
Supplement: S1 Table — (DOCX) [file pone.0120980.s001.docx]

**S1Table. Dams western/cafeteria diet information**

| Cafeteria diet | Total energy  (kJ/g) | Total fat  (g/100g) | Total protein  (g/100g) | Total carbohydrates  (g/100g) |
| --- | --- | --- | --- | --- |
| Meat Pie | 9.8 | 12.0 | 8.4 | 22.4 |
| Chocolate cake | 16.9 | 17.3 | 3.7 | 51.7 |
| Vanilla Cream Biscuits | 21.5 | 24.4 | 5.5 | 67.8 |
| Biscuits | 18.9 | 13.1 | 4.7 | 77.2 |
| Dim Sim | 7.9 | 6.8 | 5.6 | 25.8 |
| Pasta | 14.9 | 2.2 | 12.7 | 68.6 |
| Peanut butter | 26.1 | 51.5 | 27.5 | 11.3 |
