# Supplementary material for: Effects of Maternal Diet and Exercise during Pregnancy on Glucose Metabolism in Skeletal Muscle and Fat of Weanling Rats
Source: PLoS One. 2015 Apr 8;10(4):e0120980. doi: 10.1371/journal.pone.0120980 (PMC4390148; doi:10.1371/journal.pone.0120980)
Supplement: S2 Table — (DOCX) [file pone.0120980.s002.docx]

**S2 Table. Taqman probe sequence used for real time PCR**

| **SN** | **Gene name** | **NCBI gene references** | **Applied biosystem assay** |
| --- | --- | --- | --- |
| **Housekeepers gene** | | | |
| 1. | HPRT1,hypoxanthine phosphoribosyltrsnsferase1 | NM_012583.2 | Rn01527840_m1 |
| 2. | YWHAZ, tyrosine 3-monooxygenase/tryptophan 5-monooxygenase activation protein, zeta polypeptide | NM_013011.3 | Rn00755072_m1 |
| 3. | ACTB, actin, beta | NM_031144.2 | Rn00667869_m1 |
| 4. | B2M, beta-2 microglobulin | NM_012512.2 | Rn00560865_m1 |
| 5. | PPIA, peptidylprolyl isomerase A (cyclophilin A) | NM_017101.1 | Rn00690933_m1 |
| 6. | GAPDH, glyceraldehyde-3-phosphate dehydrogenase | NM_017008.3 | Rn01775763_g1 |
|  |  |  |  |
| **Gene of interest** | | | |
| 1 | GLUT4 /SLC2A4, solute carrier family 2 (facilitated glucose transporter), member 4 | NM_012751.1 | Rn01752377_m1 |
| 2. | MYOD1, myogenic differentiation 1 | NM_0176079.1 | Rn01457527_g1 |
| 3. | SIRT3, sirtuin 3 | NM_001106313.2 | Rn01501410_m1 |
| 4. | IL6, interleukin 6 | NM_012589.1 | Rn01410330_m1 |
| 5. | PGC1α/Ppargc1a, peroxisome proliferative activated receptor, gamma, coactivator 1 alpha | NM_031347.1 | Rn00580241_m1 |
| 6. | UCP3, uncoupling protein 3 (mitochondrial, proton carrier) | NM_013167.2 | Rn00565874_m1 |
| 7. | TNFα, tumor necrosis factor alpha | NM_012675.3 | Rn99999017_m1 |
